# Intermittent Preventive Treatment of Malaria in Children: A Qualitative Study of Community Perceptions and Recommendations in Burkina Faso and Mali

**DOI:** 10.1371/journal.pone.0032900

**Published:** 2012-03-06

**Authors:** Catherine Pitt, Halimatou Diawara, Dimlawendé J. Ouédraogo, Samba Diarra, Habibou Kaboré, Kibsbila Kouéla, Abdoulaye Traoré, Alassane Dicko, Amadou T. Konaté, Daniel Chandramohan, Diadier A. Diallo, Brian Greenwood, Lesong Conteh

**Affiliations:** 1 Department of Global Health and Development, London School of Hygiene and Tropical Medicine, London, United Kingdom; 2 Malaria Research and Training Centre, Bamako, Mali; 3 Department of Public Health, Faculty of Medicine, Pharmacy and Dentistry, University of Bamako, Bamako, Mali; 4 Centre National de Recherche et de Formation sur le Paludisme, Ouagadougou, Burkina Faso; 5 Department of Disease Control, London School of Hygiene and Tropical Medicine, London, United Kingdom; 6 Institute of Global Health Innovation, Imperial College London, London, United Kingdom; The George Washington University Medical Center, United States of America

## Abstract

**Background:**

Intermittent preventive treatment of malaria in children (IPTc) is a highly efficacious method of malaria control where malaria transmission is highly seasonal. However, no studies published to date have examined community perceptions of IPTc.

**Methods:**

A qualitative study was undertaken in parallel with a double-blind, placebo-controlled, randomized trial of IPTc conducted in Mali and Burkina Faso in 2008–2009 to assess community perceptions of and recommendations for IPTc. Caregivers and community health workers (CHWs) were purposively sampled. Seventy-two in-depth individual interviews and 23 focus group discussions were conducted.

**Findings:**

Widespread perceptions of health benefits for children led to enthusiasm for the trial and for IPTc specifically. Trust in and respect for those providing the tablets and a sense of obligation to the community to participate in sanctioned activities favoured initial adoption. IPTc fits in well with existing understandings of childhood illness. Participants did not express concerns about the specific drugs used for IPTc or about providing tablets to children without symptoms of malaria. There was no evidence that IPTc was perceived as a substitute for bed net usage, nor did it inhibit care seeking. Participants recommended that distribution be “closer to the population”, but expressed concern over caregivers' ability to administer tablets at home.

**Conclusions:**

The trial context mediated perceptions of IPTc. Nonetheless, the results indicate that community perceptions of IPTc in the settings studied were largely favourable and that the delivery strategy rather than the tablets themselves presented the main areas of concern for caregivers and CHWs. The study identifies a number of key questions to consider in planning an IPTc distribution strategy. Single-dose formulations could increase the success of IPTc implementation, as could integration of IPTc within a package of activities, such as bed net distribution and free curative care, for which demand is already high.

## Introduction

In the Sahel and sub-Sahelian regions of Africa, malaria transmission is highly seasonal and the main burden of malaria is in children [1], [2]. Intermittent preventive treatment of malaria in children (IPTc) is a promising new strategy for malaria control in such areas [3], [4]. Like IPT in pregnant women (IPTp) [5], [6] and IPT in infants (IPTi) [7], [8], [9], [10], IPTc consists of administration of a treatment dose of an effective anti-malarial drug (or drug combination) at predetermined time points to a specified at-risk population, regardless of parasite burden or symptoms. Despite a growing clinical literature supporting the efficacy of IPTc [4], [11], [12] and interest from policy makers in implementing this intervention, no studies published to date have examined community perceptions of IPTc or how such perceptions may facilitate or pose challenges to delivery in countries where the innovation might be deployed.

A recent systematic review and meta-analysis found that IPTc administered monthly during the high malaria transmission season reduced the incidence of clinical malaria by 82% (95%CI: 75–87%, p<0.001) [4]. While insecticide-treated bed nets (ITNs) are the malaria prevention strategy of choice in many countries [13], many children in malaria endemic areas do not sleep under a net, and even where ITN coverage is high, malaria morbidity and mortality may persist [11], [12]. To determine the added value of IPTc in children who slept under a long-lasting insecticide-treated bed net (LLIN), a double-blind, placebo-controlled, randomized trial of IPTc was conducted in Burkina Faso and in Mali in 2008–2009 [11], [12]. This trial demonstrated that even with extremely high use of LLINs (Burkina Faso: 93% [11], Mali:>99% [12]), IPTc reduced malaria morbidity substantially. In Burkina Faso, the protective efficacy of IPTc against episodes of clinical malaria was 70% (95% CI: 66%, 74%) [11] and in Mali it was 82% (95% CI: 78%, 85%) [12]. Severe malaria was reduced by 69% (95% CI: 6%, 90%) and 87% (95% CI: 42%, 99%), respectively [11], [12].

While pregnant women and infants receive IPTp or IPTi during existing health service contacts, children under five years of age do not have pre-existing contacts with the health service at the necessary intervals to receive IPTc. Thus, IPTc shares many of the implementation challenges of Vitamin A distribution, polio and measles vaccination campaigns, and mass drug administration (MDA) to combat helminth infections. However, unlike these other campaigns, current IPTc drug regimens require that children receive tablets on three consecutive days each month, as single-dose formulations are not currently available. Moreover, IPTc poses the further challenge of requiring that drugs be given during the rainy season when travel is most difficult and rural populations are focussed on farming. An effective implementation strategy requires more than mere acceptance of the intervention; local actors need to be motivated and mobilized to take an active role in implementing IPTc.

The study described in this paper set out to provide insights into the meanings community members attach to IPTc and to solicit their recommendations for its large-scale implementation. While the IPTc and LLIN clinical trial in Burkina Faso and Mali presents a somewhat artificial context, it also provides a timely opportunity to examine perceptions of the intervention before large scale implementation of IPTc is attempted in these settings.

## Methods

### 1. Study setting and conduct of the trial

Detailed descriptions of the study setting, how the trial was conducted, and its main findings have been published previously [11], [12]. In Mali, the trial was conducted at two rural health centres located 40 and 45 kilometres southwest of Bamako and at one semi-urban small town health centre 80 km south of Bamako. In Burkina Faso, the trial was undertaken in four rural health centres in Boussé District, 45 km north of the capital.

Researchers held meetings with health and local authorities to answer questions and obtain permission for the trial before meeting with women at each health facility to explain the trial, to obtain individual written consent, and to screen children for eligibility to participate in the trial. Caregivers received a LLIN for each child. Approximately 6,000 children aged 3 to 59 months who lived within 2 km of one of the seven health centres were individually randomised to receive three rounds of IPTc or placebo over three-day periods in August, September, and October 2008. Sulphadoxine pyrimethamine (SP) and amodiaquine (AQ) tablets (or placebos) were administered on the first day and AQ alone (or placebo) on the subsequent two days. Caregivers were asked to bring their children to their local health centre for supervised IPTc administration on all three days each month. Trial staff weighed children, assigned them to receive a small, medium, or large tablet, and physically administered the tablet(s) to the child by crushing them in a spoon for infants or providing sugar water and sometimes biscuits to older children. The research team paid one or more CHWs at each site to assist with the administration and to seek out and remind caregivers of children who did not attend, which resulted in very few doses missed during the trial [11], [12].

Throughout the 2008 and 2009 malaria transmission seasons, study children were monitored for episodes of malaria, hospital admission or death. Children who presented to the health centre for IPTc administration were examined and, if found to have malaria, were treated with an alternative antimalarial combination. Health care was made free at the point of use for all study participants.

### 2. Data collection

In-depth, semi-structured, individual interviews (IDIs) were conducted with caregivers of children under five years old and Community Health Workers (CHWs) to understand perceptions of IPTc at the community level and to explore local understanding of malaria prevention. These participants, as well as additional caregivers and CHWs, were invited to take part in a focus group discussion (FGD), in which participants were encouraged to formulate practical recommendations regarding future IPTc distribution in their country.

Data were collected six to nine months following implementation (Mali: May to July 2009, Burkina Faso: June to August 2009), and so reflected not the immediate response to IPTc, but rather participants' views as they approached the new rainy season.

**Figure 1:**
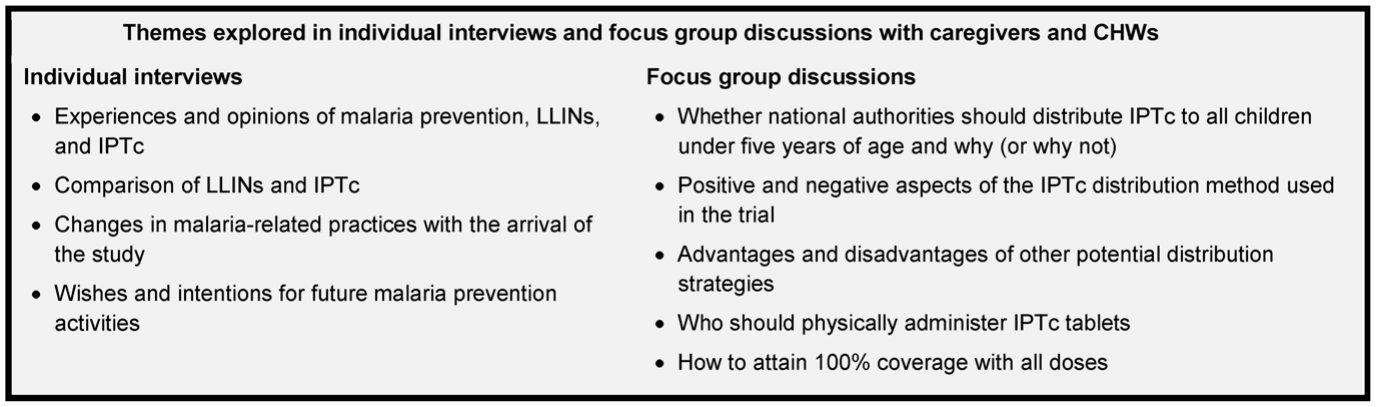
Themes explored in individual interviews and focus group discussions with caregivers and CHWs.

In both countries, interviewers included men and women with masters' degrees in sociology and experience in health-related interviewing in rural areas. In Mali, three junior physicians who had been involved in delivering IPTc also conducted interviews, although they did so only in sites where they had not provided care and were responsible for less than one-third of interviews. Interviews and FGDs were conducted in Bambara in Mali and Moré and Malinké in Burkina Faso. Themes covered in the interviews and focus group discussions are presented in **Figure 1**.

Caregiver interviews were conducted in or near the participant's home, while CHW interviews were conducted either at the participant's home or at the local health facility, depending upon the CHW's preference. FGDs were conducted at or near the health facility. At each FGD, a single interviewer facilitated the discussion, while a second observed and took notes, and a third observed and took care of practicalities. Interview participants indicated that mixed sex FGDs would be culturally inappropriate for caregivers but that mixed sex FGDs with CHWs were acceptable.

### 3. Sampling

The sample size was set at approximately 90 to 120 interviews and FGDs across the two countries *a priori* on the basis that this would prove neither so large a sample as to inhibit deep, case-oriented analysis nor so small a sample that it would become difficult to achieve data saturation, theoretical saturation or information redundancy [14], [15], [16].

Participants were purposively selected from amongst the caregivers involved in the trial and from amongst the CHWs associated with health posts involved in the trial with the aim of including a wide range of views and experiences associated with IPTc and malaria prevention. The caregiver population was initially defined as the primary female caregivers of all children under five years of age who were eligible to participate in the trial. However, preliminary interviews highlighted the key role of male relatives in decision making around child health, and so a smaller proportion of self-defined male “caregivers,” was included in the overall sample. The caregiver sample was stratified to include three levels of participation in the trial: highest participation (child received all 9 doses without any reminder), moderate participation (child missed 1–2 doses, or received all doses only after repeated reminders), and lowest participation (refusal, withdrawal, or multiple missed doses). Active or placebo status could not be included in the sampling criteria as epidemiological data collection was ongoing and researchers were still blinded as to which study group a child belonged. The CHW population was defined as both men and women of varying ages already working as CHWs within the catchment area of each of the health centres included in the trial. CHWs were selected from villages included in the IPTc efficacy trial and those from more distant villages which were not included in the trial.

If an identified person refused or was not available to participate, a replacement from the same sub-group with similar characteristics was identified. At the end of each individual interview, the participant was invited to take part in a group discussion at a specified time and place later in the week.

### 4. Data entry and analysis

Data from each interview and each FGD were prepared in a standardized format, which included basic socio-demographic information, the interviewer's own observations and reflections on the interview, and the verbatim transcript in French based on digital audio recording(s). The FGD facilitator and the two interviewers who had observed the FGD collaborated to prepare the FGD transcript, observations, and participant information. In most cases, the transcript and observations were prepared within 24 to 48 hours of conducting the interview or FGD.

All data were uploaded into QSR NVivo 8.0 ©. The French-language transcripts were coded line by line using detailed themes and sub-themes identified *a priori* as being relevant to the initial research questions, as well as using detailed themes and sub-themes that emerged from the data, including the interviewers' observations, and from theoretical and empirical literature. Attributes such as country, sex, number of children in the trial and number of doses each child received were associated with each interview and (in most cases) FGD participant. Active or placebo status was revealed and employed in the analysis only after thorough coding. The frequency and content of sub-themes were assessed for similarities or differences across different attribute values, for example comparing the frequency of reporting side effects amongst children who received active IPTc compared with those who received placebo. Illustrative quotations have been translated into English and are included in the results.

### 5. Ethical considerations

Both the clinical trial and this qualitative sub-study were approved by the ethics committees of the relevant research institutions in the UK (London School of Hygiene & Tropical Medicine), Mali (Malaria Research and Training Center), and Burkina Faso (Centre National de Recherche et Formation sur le Paludisme).

Written informed consent was obtained from all participants before participation in interviews and focus groups and was reconfirmed orally before digital audio recording. For those who participated in both an interview and a FGD, consent was obtained separately for each. Where participants had difficulty reading a full page of text, a member of the research team read out the consent form and a literate community member unconnected with the study (usually the local school teacher) acted as an independent witness of the consent process.

## Results

Seventy-two interviews and 23 FGDs were conducted with 179 participants (**Table 1**). Twenty-seven of the 58 CHWs who participated in an interview and/or FGD (47%) also identified themselves as a caregiver of at least one child who had participated in the trial; they are categorized as CHWs rather than caregivers. The trial status of the children of these CHWs is shown in **Table 2**. Interviews lasted an average of 30 minutes and FGDs lasted approximately one hour.

**Table 1:** Number of individual interviews, focus group discussions (FGDs), and participants by country and sex of participant.

			Mali		Burkina Faso		TOTAL
			Female	Male	Female	Male	
CHWs	Individual Interviews		5	7	2	10	24
	FGDs	Groups	4 (mixed sex)		4 (mixed sex)		8
		Participants	10	16	8	20	54
Caregivers	Individual Interviews		17	7	15	9	48
	FGDs	Groups	4	3	4	4	15
		Participants	29	23	24	24	100
**TOTAL**		Transcripts^a^	47		48		95
		Participants	93		86		179^b^

a The total number of transcripts is the sum of individual interviews and focus groups conducted.

b The total number of participants in the study (n = 179) is less than the sum of participants in individual interviews (n = 72) and participants in the FGDs (n = 154) because some (n = 47) participated in both an individual interview and a FGD.

**Table 2:** Trial status of the children of participating caregivers.

	IPTc status of participant's child(ren)	CHWs**		Caregivers		TOTAL	
**Mali**	All received active IPTc	3	(30%)	21	(32%)	24	(32%)
	Mixed – at least one received placebo and one active	4	(40%)	14	(22%)	18	(24%)
	All received placebo	3	(30%)	19	(29%)	22	(29%)
	Refused	0	(0%)	9	(14%)	9	(12%)
	Status unknown	0	(0%)	2	(3%)	2	(3%)
**Burkina Faso**	All received active IPTc	5	(29%)	24	(43%)	29	(40%)
	Mixed – at least one received placebo and one active	5	(29%)	16	(29%)	21	(29%)
	All received placebo	7	(41%)	16	(29%)	23	(32%)
	Refused	0	(0%)	0	(0%)	0	(0%)
	Status unknown	0	(0%)	0	(0%)	0	(0%)
**TOTAL**	All received active IPTc	8	(30%)	45	(37%)	53	(36%)
	Mixed – at least one received placebo and one active	9	(33%)	30	(25%)	39	(26%)
	All received placebo	10	(37%)	35	(29%)	45	(30%)
	Refused	0	(0%)	9	(7%)	9	(6%)
	Status unknown	0	(0%)	2	(2%)	2	(1%)
**TOTAL**	27	121	148				

** CHWs without children eligible for the trial are excluded from this table.

The trial context was found to play an important role in mediating perceptions of IPTc. Therefore, we focus first on perceptions specific to the trial context.We then explore five themes which, while mediated by the trial context, would also be relevant for future IPTc implementation: perceptions of IPTc tablets and side effects, their compatibility with existing understanding and experience of childhood illness, the social context of participation, and perceptions of tablet distribution during the trial. Finally, participants' own recommendations as to how IPTc should be distributed in future are presented.

Results from the two rural sites in Mali and four rural sites in Burkina Faso were strikingly similar. The most notable differences were not between the two countries, but rather between the six rural sites and the single, semi-urban site. Differences in perceptions based on these and other participant characteristics are highlighted where they emerged.

### 1. Interpretation of IPTc as part of a package of trial activities

Perceptions of the trial, which was known locally as “the project” or “the malaria project”, were overwhelmingly favourable. Participants tended to conceive of the trial as a single entity. Opinions based on experiences of one trial-related activity, such as provision of free curative care or LLINs, were sometimes directly attributed to another activity or to “the project” or “the malaria project” as a whole. Aspects of the trial which were specific to the trial context thus influenced overall perceptions of “the project” and even perceptions of the IPTc tablets and their distribution.

Two aspects of the trial engendered concern amongst some participants at the start. In the semi-urban site in Mali, the very concept of a trial seemed to raise concerns, as rumours spread that the IPTc tablets would cause infertility, that the medicines were expired, and that the research team would sell children's blood. A few residents also seemed to have been under the (false) impression that a new drug was being tested. Nearly all of the small number of eligible families who refused to participate in the trial and 29 who formally withdrew consent were in this town, the only non-rural trial site in the study [12]. Men appeared to play a significant role in these decisions. While this town had experience of other trials, so too did some of the other sites; the key difference was the town's far greater socio-economic diversity. An educated local leader refused to allow his children to participate and actively discouraged his neighbours.

*… the children started taking the medicines, and he came and said ‘you mustn't accept, you, you're educated, and your children will be guinea pigs.’*

*–Female caregiver, FGD, Mali, mixed active and placebo (ML FGD08-P3)*

*Well, really for a farmer, if you're told that a new medicine has come, that they're going to test it on you, that's a problem. That's why a lot of people withdrew.*

*–Male caregiver, FGD, Mali, refusal (ML FGD11-P6)*

Yet, after observing the trial over the course of the malaria season, many of the caregivers who had refused or withdrawn their children and those whose children had not been eligible to participate actively lobbied “to be included in the project.”

The references in the consent forms to the need for finger-prick blood samples also raised some concern in all the sites, although these did not seem to lead to refusals or withdrawals. While many caregivers and CHWs described blood samples in a neutral or positive light, explaining that the “doctors needed to look in the blood for malaria,” some caregivers were concerned that the blood would be sold or that the blood loss would make their child “weak” or “bring another disease.” These fears over the quantities of blood that would be taken suggest that some believed that participation in the trial would require the collection of larger samples through venipuncture.

Two other aspects of the trial, the provision of free LLINs and free curative care for trial participants, contributed positively to perceptions of the trial. When the trial team distributed LLINs at the start of the malaria season, many women arrived at the health centre in the middle of the night to ensure that they would not miss out, demonstrating the high value placed on this “gift” despite low levels of prior usage. In expressing enthusiasm for IPTc or for “the project” in general, participants often explained the advantages in terms of the benefits of the free curative care.

*What the tablets have brought in terms of benefits can't all be cited. It helps families in different ways. Someone can be sitting there without 100 francs [$0.21, 2009 USD] and gets his child treated for free. The cock that you were going to sell to treat your child, you can keep.*

*– Male CHW, IDI, Burkina Faso (KK00)*

The trial team encouraged prompt care seeking, but many participants specifically cited the removal of this financial barrier as increasing their utilization of curative health services. In Mali, where research team doctors were on site 24-hours a day, participants particularly appreciated the willingness of the doctors to provide care at any hour “nicely and with respect” and without charging fees.

### 2. Perceived impact and interpretations of IPTc tablets and side effects

When asked specifically about “the tablets your child received nine times last year in the malaria season, three times in a row in August, three times in a row in September, and three times in a row in October,” opinions were very favourable. Most participants commented on reductions in morbidity and mortality, but these positive responses were evident amongst both those caring for children who had received the placebo and those whose children had received the active drug. None reported observing differing levels of effectiveness of the tablets for different children nor did they attribute differences to placebo tablets. Indeed, in all the interviews and discussions conducted, only one person, a CHW, alluded to the fact that half the participants received placebo tablets. Nonetheless, caregivers of children who had all received active IPTc were considerably more likely to attribute the morbidity reductions specifically to IPTc, whereas those whose children received placebo were more likely to attribute benefits more vaguely to “the project.”

*… since mine received the tablets, he has not fallen ill any more. He hasn't even complained of headaches.*

*–Female caregiver, IDI, Burkina Faso, active IPTc (KH04)*

*The tablet that was given to children, ha, people have really seen a lot of benefits from it. We have really seen benefits from it, because at the time of the administration, my child didn't fall sick. We took all the three doses, my child hasn't had malaria, we haven't found any dizziness.*

*–Female caregiver, IDI, Mali, active IPTc (BS04)*

The absence of illness and the presence of “good health” was a widely observed (non-)event. While caregivers appreciated the decrease in child illness, they also appreciated its positive consequences for themselves.

*… here now in Djoliba malaria has decreased. I wouldn't say that it is completely gone, but, if it pleases God, we thank God, the number of deaths of children has decreased a lot. In the past, we couldn't even farm because of the deaths.*

*–Male CHW, IDI, Mali (FD01)*

*The tablets have spared us from many worries. Thanks to the tablets, we no longer take our children to the health centre because they are no longer sick. We can also take care of our field work.*

*–Male caregiver, IDI, Burkina Faso, mixed active and placebo (OD16)*

Most caregivers did not offer any particular explanation of how IPTc worked, but a few caregivers and many CHWs described IPTc as “fighting” against invisible “germs of disease” in children's bodies. Only a small proportion of caregivers referred to IPTc as vaccination or immunization:

*I took my child for the malaria vaccination because … malaria is a very contagious disease that can kill in an instant … even if you don't take the medicine [IPTc], if you are […] protected by the bed nets against mosquitoes, it's very likely that you won't get sick with malaria.*

*–Female caregiver, IDI, Mali, placebo (SD13)*

Questions did not specifically seek opinions on the drug combination, SP and AQ, but participants did not raise the issue either, suggesting that opinions of IPTc were not linked to pre-formed opinions about the IPTc drugs.

During the consent process for the trial, caregivers were warned of the potential side effects of IPTc, which included vomiting, diarrhoea, and fever, and for which they could receive treatment at the health centre. For some, this warning was reassuring, and so they were not alarmed by the side effects when they occurred, while others were concerned or bothered enough about the side effects that they were reluctant to return to the health centre for subsequent IPTc doses. These responses related to how the caregivers interpreted the side effects. Several CHWs and caregivers described side effects as the product of the interaction between the IPTc drugs and disease in the body – and therefore not as a risk, but a positive sign that the medication was “working” – which was a common understanding of the action of traditional remedies. A few caregivers explained that children need to “get used to” IPTc.

*P7: Although they were told about the reactions the medicine can cause, certain mothers refused to give the medicine to their children. They say that it causes this and that to my child, although we were well informed about that before the start of the project. It's written for those who can read, and repeated to those who don't know how to read, but with all that some people were afraid. But after the benefits were seen, it cured malaria in a lot of children.*

*P2: Me, I didn't see these reactions in my children, no vomiting, no diarrhoea.*

*P3: Even if the child vomits, you are asked to bring the child to the centre.*

*P2: Because if you are having the treatment and when the medicine arrives at the disease, it causes reactions, so that's what provokes diarrhoea and vomiting.*

*–Female caregivers, FGD, Mali (ML-FGD02)*

*Any food that you're not used to, if you eat it before your intestines get used to it, it hurts you. And, it's the same with medicines for children. We are given the medicines, if the children have diarrhoea, they say “don't hesitate, come have the anti-diarrhoeics” … this is how as they go along the children get used to the medicine.*

*–Female caregiver, FGD, Mali, placebo (ML-FGD02-P6)*

The caregivers interviewed were more likely to report side effects in Mali than in Burkina Faso, though reports of side effects were uncommon overall. Caregivers were equally likely to report that their child or children had experienced side effects regardless of whether the child had received active or placebo tablets. These results appear to contradict the quantitative results reported in the trial papers, which found that vomiting was more frequent in children who had received IPTc and far more frequent in Burkina Faso than in Mali (27.6% vs. 4.0% of children receiving active IPTc vomited at least once) [11], [12]. One explanation for this anomaly may be the purposive rather than statistically representative sampling used in the qualitative data collection. It is also possible that caregivers whose children received active IPTc were less likely to report negative aspects of IPTc, either because their positive overall assessment of IPTc coloured memories or interpretations of side effects many months before, or because they did not want to say anything that might negatively affect future IPTc implementation.

### 3. Compatibility of IPTc with existing understandings and practices

IPTc in itself was compatible with existing understandings, practices, and experiences of childhood illness. Participants' syncretic approach to health meant that adopting IPTc did not mean rejecting existing practices or understandings; IPTc could, in effect, be “added” without necessarily displacing existing practices.

Most participants identified mosquito bites as a cause of malaria. As found in previous studies [17], [18], CHWs and caregivers in both countries also attributed malaria to dirty hands and food, certain (cold) foods, and divine will. When asked what they do to prevent malaria in their children, a few caregivers explicitly said that prevention was not possible, but most cited a number of both preventive and curative strategies, including bed nets, “hygiene”, insecticides, “taking the child to the health centre when he is ill,” and wrapping children warmly. The study communities repeatedly and spontaneously cited their past experiences with chloroquine chemoprophylaxis (known by its brand name, “nivaquine”), with which they were very familiar:

*… we were told not to give nivaquine to children, but in the past, I used to buy a lot of nivaquine during the rainy season. I kept it at my expense. Each time I used to give these nivaquines to the children until the dry season. That's what used to keep them from falling ill.*

*–Male CHW, IDI, Mali (BS01)*

Many caregivers were, therefore, already familiar with using tablets both to treat and to prevent malaria and commonly employed a number of different malaria control strategies simultaneously.

Some people described in detail their conception of the complementary nature of LLINs and IPTc:

*I think that the two methods used together is better, because in sleeping under the bed net, the child can find himself outside of the bed net and thus expose himself to mosquitoes. But, if the bed net is accompanied by a tablet, the body is immunized to fight against the germs of disease.*

*–Male caregiver, IDI, Burkina Faso, placebo (OD01)*

IPTc was thus seen as highly compatible with and complementary to LLINs; there was no evidence that people perceived them as substitutes. While this attitude is likely to have been partly a reflection of the research team's sensitization messages encouraging bed net usage, it also seemed to fit in with a more general perception of the need for new and better strategies to combat malaria. In discussing the benefits of IPTc, several participants also explicitly expressed openness to any strategy that could prevent malaria or improve health.

*The last words I have to say are that really, this medicine [IPTc] is very important. I can't say how important it is. Since it was given to us, there has been good health. We really feel that a lot, we like this medicine a lot. If another one is found in addition, that's what we're looking for.*

*–Female caregiver, FGD, Mali, IPTc status unknown (ML-FGD04)*

While participants did not clearly distinguish preventive from curative activities, they did draw a clear distinction between “modern,” “Western” or even “white people's” medicine and “traditional” medicine. Very rarely did participants cite traditional practices initially, but when prompted specifically, and sometimes when asked about traditional preventive practices that “other people” use, they cited placing leaves in hot water for the sick child to bathe in, drink, or inhale the vapours and burning the shells of Shea nuts or, less frequently, cow dung, to fill the house with smoke and chase mosquitoes away. Some said that “times have changed” and that traditional medicines are no longer effective because children “have become modern”, because “people's blood isn't the same now”, or because “people's food has changed now”, but none of the participants indicated that use of one type of preventive activity precluded use of another.

### 4. Participation in IPTc as a social act

Perceptions of and participation in IPTc were also shaped by several key aspects of the social context, independently of personal views and experiences of IPTc or the trial. Trust in and respect for authorities, including formal health workers, emerged as an important motivator for adopting IPTc. Older participants in Burkina Faso in particular described the distribution of IPTc and LLINs as “a gift,” citing the importance of accepting and the dishonour to the community of refusing a gift. Both caregivers and particularly CHWs described participation and adherence in terms of “obeying” or “disobeying,” suggesting a sense of obligation to comply. There was no evidence that the trial team coerced participation, but caregivers and CHWs both seemed to place a significant social value on respecting hierarchical social structures, which were seen to have sanctioned IPTc and the trial as a whole.

*As it's the nurses who gave it, it's required that you use it. If you take the bed net and you say that you will no longer go to the health centre [for the IPTc tablets], that, too, is not right.*

*–Female caregiver, IDI, Burkina Faso, placebo (KK02)*

*… I accepted because they cannot come here without going through our authorities to explain it to them. And we are under them [the authorities], and someone who is your authority, if he sends you someone who is going to care for you, this latter person must know something […] He's not going to send you an idiot.[… ] Our authorities love us …*

*–Male caregiver, IDI, Mali, placebo (SD08)*

The degree to which participation was an observable social activity also seemed to encourage participation.

*P1: Me, all my neighbours came.*

*P5: We come as a group. When one woman goes out, she calls the others and we come to the health centre together.*

*–Female caregivers, FGD, Burkina Faso (BF-FGD01)*

One man who had initially refused for his children to participate was reassured regarding the safety of IPTc by the observation that wealthy community members were participating.

*My case is a lesson and advice … If it causes sterility, the rich people are signing up their children because they don't love their children? They love them! . . when I realized the lie, I ran [to be included], but you [the study team] refused. I can't force you, but I would like to enter [the trial].*

*–Male caregiver, IDI, Mali, withdrew from trial (SD09)*

While sensitization activities focussed on women, male caregivers emerged as playing an important role in decision-making in both countries, whether they were for or against participation. Several of the cases of refusal or withdrawal were linked to a male family member, rather than the mother.

*It's true that the child belongs to both of you, but as a woman, you can decide to take the child to the health centre and your husband wants you to go to the fields. If you disobey and you go to the health centre, your husband will say that you disobeyed him and you will have difficulties to come back home… it doesn't make you happy, but you resign yourself to your husband's will … If the child is sick you can take him quickly to the dispensary without your husband's authorisation. Other than this urgent case, men don't accept.*

*–Female caregiver, FGD, Burkina Faso, IPTc status unknown (BF FGD01)*

*It was a bit difficult for me because people went and told my father-in-law bad rumours, that this work, this thing, wasn't good… He told us to abandon this thing [the trial] … That's why we didn't continue the rest of the trial.*

*–Female caregiver, FGD, Mali, mixed active and placebo (ML-FGD06-P1)*

*The child's father said that if the child is not included in this thing [the trial], he is going to divorce me.*

*–Female caregiver, FGD, Mali, mixed active and placebo (ML FGD08-P4)*

### 5. Perceptions of IPTc delivery in the trial

Many male and female caregivers expressed satisfaction with distribution of IPTc at the health centre. One advantage that emerged was that it provided an opportunity for contact with a health worker, which both instilled confidence in IPTc and gave the opportunity for children to be seen by a health worker.

*Because even we women, there are women who have children, but they don't know that their children are sick. But there, if a woman brings her child and the health workers look at it, they can detect the child's illness.*

*–Female CHW, FGD, Burkina Faso (FGD06-P6)*

In a number of cases, participants also compared the effort of taking their children for IPTc administration with the consequences of them falling ill and the importance of child health.

*We know that if we're told to evacuate him to [the district hospital at] Boussé once, it's really not easy. So,[taking IPTc tablets] 9 times is easier than Boussé once.*

*–Male caregiver, FGD, Burkina Faso (BF-FGD02)*

*P1:… health is worth more than anything. It's true that it's the season for [field] work, but it's because there is health that we can work -*

*P5: - [interrupting] it's true, if there is not health, we cannot work […]*

*P4: If your child is sick, you take him to the health centre even if it's in the harvest season. Too bad for the field work. To preserve your child's health, you must not worry about field work. The health of the child is the priority.*

*–Female caregivers, FGD, Burkina Faso (BF-FGD01)*

The most common reason for a missed dose in both countries was that the child had travelled with his mother to another area, or that the person who would normally take the child to the health centre had travelled, even though the child was still at home. In addition, many children were not included in the trial because their family intended to travel, which was especially common amongst nomadic Peuhls in Mali and amongst migrant labourers in Burkina, who often travelled to Côte d'Ivoire.

Although only those families living fewer than 2 km from the health centre were included in the trial, the most frequently cited areas of dissatisfaction with the distribution of tablets were the time spent travelling to and from the health centre, and the time and experience of waiting at the health centre for many hours, often in the sun, on the distribution days. These were cited as reasons why some children missed doses during the trial and why some caregivers only came after repeated reminders.

*The method of taking [the tablets] was difficult because the day they told us to come, there were a lot of people who came out and filled the area all around the health centre. Nobody was able to work that day, you understand. There were fights and insults.*

*—Female caregiver, FGD, Mali, IPTc status unknown (ML-FGD04)*

*Of course it's difficult! [shouted] It's not easy to put a child on your back and to carry the other to the health centre. If you don't eat and you must carry two children and walk to the health centre, it's tiring. When you arrive, you feel dizzy. [laugh] After, you're accused of refusing to respond to the convocations.*

*–Female caregiver, IDI, Burkina Faso, placebo (KH11)*

Women who were the only woman in their household found it particularly difficult to wait for IPTc each day because they needed to return home to prepare the family's meal. A number of caregivers reported asking friends or neighbours to bring their children, but they were not always successful. In Mali in particular, women who were reluctant to seek IPTc at the health centre were often described as “lazy” or lacking in “courage”:

*P4: Mothers know the importance of the treatment, but they are lazy, they are not courageous –*

*P7: They know the importance of the treatment, but leaving their fieldwork, especially the groundnut harvest, in favour of the child's treatment, that's difficult. And then with the approach of the harvest …*

*–Female caregivers, FGD, Mali (ML-FGD02)*

### 6. Participants' recommendations for IPTc distribution in future

In FGDs, caregivers and CHWs discussed a number of strategies to ensure that all children in the catchment area of each health post, including those living in more distant villages, would receive all nine doses of IPTc if the health service were to decide to distribute it in future. Key issues that emerged included sensitization, the location of distribution, the role of formal health workers and CHWs, and the role of caregivers.

To overcome initial scepticism regarding a new intervention, participants in both countries recommended that information should be provided well in advance by radio and in person by CHWs. Many focussed on the visible presence of health workers, and in some cases community leaders, as an important factor instilling confidence in the intervention.

Many CHWs and caregivers in both countries argued that distribution needed to be “closer to the population” than in the trial. While a few participants favoured continuing distribution only from the health post and others recommended a door-to-door strategy, distribution from a fixed location in each village or neighbourhood was supported most widely, especially if those living nearby could continue to receive IPTc from the health post. Participants preferred fixed locations in each village because CHWs would not waste time going to homes when people were not there, caregivers could stop by at a time of day convenient for them, and it would be easier to organize. One CHW also advocated fixed distribution points to prevent fraud:

*It's so that it will be in front of everyone's eyes! Because we all who are sitting here in one place, some will verify papers, attendance, and other will give the tablets! Like that, there can't be any fraud!*

*-Male CHW, FGD, Burkina Faso (BF-FGD00-P1)*

Another CHW suggested combining fixed location and door-to-door distribution:

*… if we find that there are people who haven't come, we can find them at home to give them the tablets!*

*–Male CHW, FGD, Burkina Faso (FGD06-P2)*

Preferences for a particular distribution strategy were closely associated with perceptions of the appropriate role of formal health workers and CHWs. Many caregivers and CHWs in both countries considered a key advantage of distribution from the health centre or from a fixed point in each village to be that it would allow for the visible and reassuring presence of a formal health worker. Some caregivers in both countries expressed their lack of confidence in CHWs particularly strongly, although a CHW in Mali indicated that willingness to follow CHWs varied between areas.

*P4: … if a CHW sets up in my neighbourhood to give the tablets without the assistance of a health worker, I will refuse these tablets. I won't have confidence! […]*

*P6: We who are close, we can come to the health centre to take the tablets with health workers. For those who are far away, it would be better if a CHW and a health worker go do the distribution. There are a lot of illiterates amongst the CHWs. Without the help of a health worker, it would be difficult …*

*–Male caregivers, FGD, Burkina Faso (BF-FGD08)*

*Well, as we are from the [rural] village, I can say that here [in the town centre] it's more difficult than for us. Because if you say to people to come out to take your medicine on this day, the day you say, it's very unlikely that people will miss this day … there are a lot of problems in town, but with us in the bush, it's not the case.*

*–Male CHW, FGD, Mali, (ML-FGD05-P3)*

Regardless of their preferred strategy for delivery of IPTc, both CHWs and caregivers emphasized the importance of training the CHWs for their tasks. Discussions with CHWs revealed that most did not have experience in or the equipment for weighing children, although they were familiar with measuring height for dosing albendazole as part of MDA to combat helminth infections.

Several CHWs suggested that if children were enumerated in advance, as was done in the trial, coverage would be higher and distribution would be simpler, regardless of the distribution method selected.

*The CHW needs to have a list with all the information about each child: his weight, his height, the dose of tablet that he needs. The following day and the day after, it will be easy for the CHW to give the remaining doses to the child.*

*–Male CHW, FGD, Burkina Faso (BF-FGD09-P3)*

The question of who should administer AQ tablets on the second and third day each month elicited the firmest opinions. The vast majority of male and female caregivers and CHWs were against proposals to leave the two AQ tablets with the child's caregiver to administer on the second and third days. Instead, they proposed that either a CHW or a health worker should administer these doses. The most common reasons given were that the caregiver would forget or would not be able to administer the tablet, but a number of participants also raised concerns about how caregivers could re-administer tablets at home if their child vomited or spat out the tablets. A few participants also cited doubts about caregivers' ability to store tablets correctly.

*If it's given to people, some may not give it, but if you ask them, they may say out of shame that they've give it when they haven't give it … Thus, if the nurses or CHWs give it at fixed points, that's better than giving [the tablets] to parents.*

*–Male caregiver, FGD, Burkina Faso (BF-FGD02).*

*P5: Well, the children are also more afraid of doctors than their parents. If parents put them [the tablets] in their mouths, they may refuse. But if it's doctors, they won't. And the medicine will be wasted -*

*P1: - and the distribution of albendazole that we do here, you yourselves, you see them vomit and do everything, but even if they vomit they're given more until they swallow. What mother can handle that with her child?*

*P5: All mothers love their child. If the child refuses to swallow, that's it –*

*P3: It's not a question of loving, it's not that, it's –*

*P1: It's courage.*

*P3: Everyone wants her child to get well. If it's said that this medicine cures him, you give it, but they are not courageous… it's not easy.*

*P5: They're not courageous.*

*–Female caregivers, FGD, Mali (ML-FGD08)*

Participants cited caregivers' experience in giving tablets at home to treat illness both in support of and against asking caregivers to administer IPTc at home.

*… if the child has a fever, they give him [the medicine], but once the child is better, the woman forgets the rest of the treatment [said while banging his hand on the table for emphasis]*

*–Male caregiver, FGD, Mali, refusal (ML-FGD09-P1)*

*For the two following days, the parents can give the tablets because they're used to giving tablets at home when the child is sick. But if the child vomits the tablet, that's the problem!*

*–Female caregiver, FGD, Burkina Faso, IPTc status unknown (BF-FGD10)*

Opinions were more evenly divided as to whether mothers or fathers should be relied on to provide these doses; while women and men each generally considered themselves more capable, many agreed that both men and women should receive information on how to give the tablets.

## Discussion

**Figure 2:**
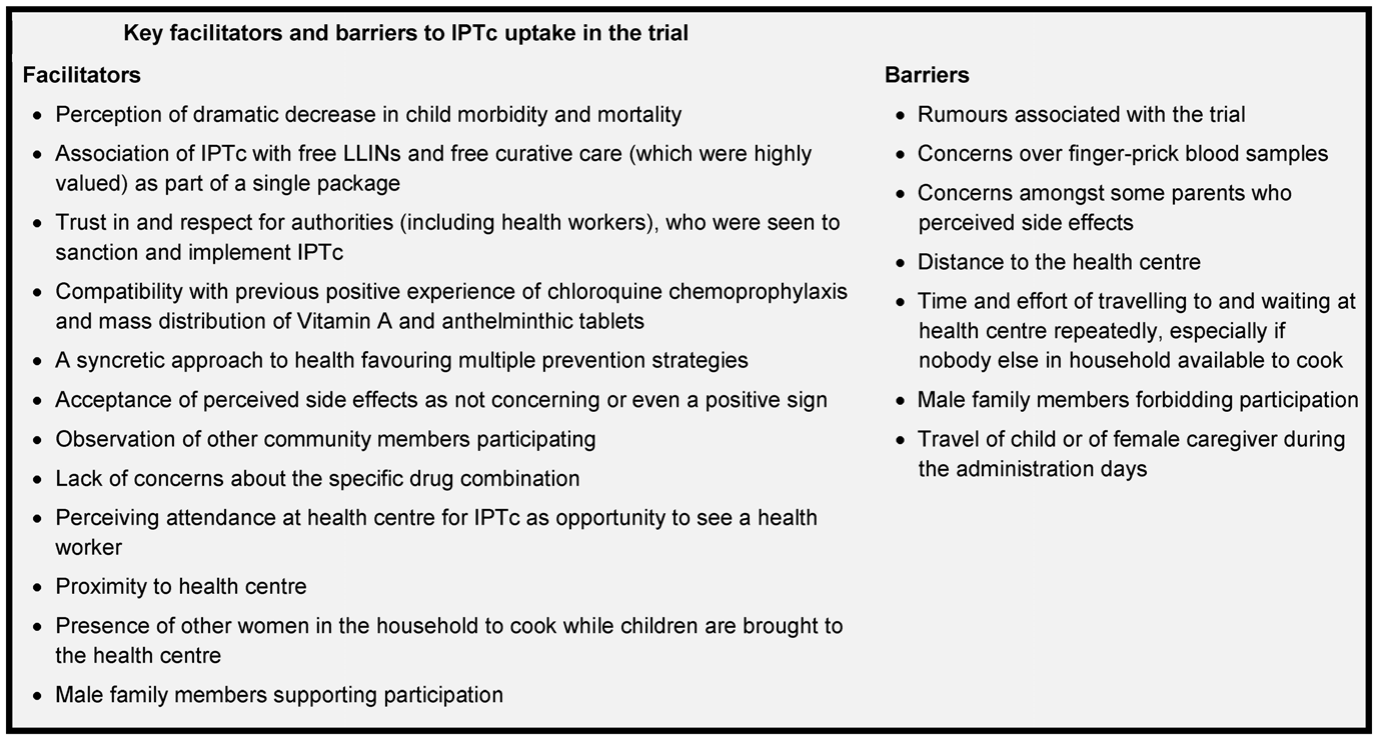
Key facilitators and barriers to IPTc uptake in the trial.

**Figure 3:**
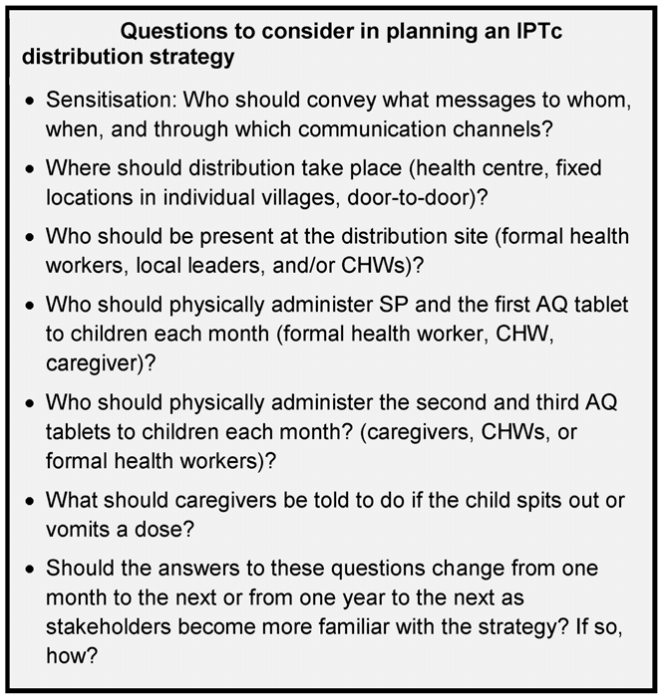
Questions to consider in planning an IPTc distribution strategy.

While Rogers finds that preventive innovations tend to diffuse slowly because their relative advantages are difficult to see [19], participants in this qualitative study quickly observed the absence of malaria in children participating in the trial. This perceived reduction in morbidity was not always attributed specifically to IPTc, but sometimes to the trial more broadly. However, it emerged as an important driver of participation, and adherence to IPTc was very high. **Figure 2** summarizes the main barriers and facilitators to uptake of IPTc in this trial. Results from the two rural sites in Mali and four rural sites in Burkina Faso were strikingly similar; the most notable differences were between the six rural sites and the single, semi-urban site. Many of the themes that emerged echoed those identified in recent studies of the acceptability of IPTi in six African countries [20] and of the acceptability of IPTp and intermittent screening and treatment (IST) during antenatal visits in Ghana [20]. This study presents findings from francophone West Africa, which was not included in either study of IPTi or IPTp, broadening the scope of investigations into the acceptability of IPT. While the results of this study cannot be used to reach generalized conclusions about how IPTc should be delivered in all settings, **Figure 3** highlights several aspects of IPTc implementation that are modifiable, of importance to community members, and likely to affect coverage.

A number of contextual factors favoured uptake of IPTc in the trial. The intervention was compatible with perceived needs and its effects were observable because of the high efficacy and uptake of the intervention and the high (perceived and real) reduction in the incidence of malaria. The rate of adoption of IPTc might, therefore, be lower in settings where the incidence of malaria is lower or where caregivers are satisfied with their existing malaria control strategies. IPTc also resonated with previous experience in these sites of chloroquine chemoprophylaxis and anthelminthic MDA, and so the concept of providing tablets to children without clinical symptoms of malaria did not raise concerns. IPTc fits in with ideas around the importance of children's health and multiple malaria aetiologies. Other studies [17], [18] have suggested that the idea that malaria may have multiple aetiologies can lead people to believe that LLINs will not be fully protective; this misconception may in fact aid complementary uptake of IPTc without reducing use of bed nets. Indeed, a syncretic approach to health in other African settings has been shown to promote complementary use of multiple disease prevention strategies, such as vaccination and traditional talismans [21], [22], [23], [24]. While this approach to health may affect treatment choices [21], [23], [24], there was no evidence that use of IPTc would inhibit care-seeking. In common with the findings from IPTi studies, which reported that some mothers viewed the side effects of vaccines as signs that they had “worked” [20], we also found that some caregivers interpreted side effects of IPTc as a positive sign that the treatment was effective “when the medicine arrives at the disease”, or as a sign that the child needed to “get used to” IPTc. In contrast to findings in the IPTi studies [20], [25], [26], participants did not express any concerns about the specific drug combination used.

Social norms and structures in both countries encouraged trust in, respect for, and compliance with authority figures in general and health staff in particular. This facilitated initial willingness to try IPTc, when none of the community members had prior personal experience of this approach to malaria control. The initial refusals and withdrawals in Mali, though small in number, were concentrated in an area of greater socio-economic diversity with more educated residents, and seemed to reflect a degree of scepticism regarding the trial, which could also affect willingness to try a new intervention. This motivation to comply with sanctioned health activities also emerged in the studies of IPTi [20] and in the comparison of IPTp with IST [27]. A growing literature around the ethics of trial participation has explored the important role of social relations in trials [22], [28], [29] and found that “interactions and relationships between researchers and community members, and within the community, play a critical role in participants' perceptions of a study” [28]. Outside a trial context, other authors have noted that a sense of “group citizenship” [30] and respect for authority can motivate participation in health campaigns. In such cases, participation is related less to the perceived characteristics of the innovation itself and more to the intangible social benefits of participating and being seen to participate. The generalizability of our results and the speed of initial adoption of IPTc would, therefore, likely be closely associated with the level of trust and respect for the institution and individuals seen to implement the intervention and the extent to which caregivers felt a sense of obligation to their community to participate.

The study highlights a number of measures that could facilitate uptake of IPTc at scale. The distribution method used in the trial, which required all participants to attend the health facility on three consecutive days each month for three months, would not be feasible for children living in more distant villages and was problematic for some of those living even quite close to the health centres involved in the trial. A small-scale study in Ghana compared delivery of IPTc at fixed locations in villages with a facility-based delivery strategy and concluded that “to maximise the impact of IPTc, both delivery systems may be needed in some settings.” [31] In The Gambia, a larger study found that delivery through CHWs was both more effective and less costly than delivery through monthly outreach visits by a team of health workers [32]. In both these studies, as well as in a large-scale study in Senegal, caregivers were asked to administer the second and third doses of AQ to their children at home, while in our study, both caregivers and CHWs in Burkina Faso and Mali expressed concern over such an approach and said that they would prefer that a CHW or health worker administer all tablets. Further studies will be needed to compare the most appropriate distribution strategy for a given context, both initially, and once community members and health workers have become familiar with the new strategy. As also highlighted in a study of IPTi [20], development of appropriate single-dose formulations for IPTc would greatly facilitate administration and would likely permit higher, more equitable, and more cost-effective coverage of the intervention. Measures may also be needed to address the challenges of highly mobile populations, especially if IPTc were to be administered in some areas and not in others.

Enthusiasm for IPTc was high in the trial in part because of its association with distribution of free LLINs and of free, high-quality, curative care, as noted in other trials [20], [22], [27], [29]. From the perspective of most caregivers and CHWs, “the project” consisted of more than simply receiving IPTc; it encompassed the entire package of trial activities, including IPTc, free LLINs, and free high-quality curative care, as well as blood tests and home visits for some families. In the language of the theory of innovation diffusion, “the project” therefore constitutes a “technology cluster” [19] comprised of several interrelated innovations. A similar introduction and marketing of IPTc as part of a package of effective strategies could facilitate adoption of all elements of the package. While scaling up any one of these strategies presents a range of challenges [33], [34], [35], uptake and enthusiasm for IPTc alone could be lower if it were introduced separately from free LLINs or free curative care, for which demand was already very high.

Caregivers in a real-world context could also perceive a greater relative advantage to taking IPTc than seen in this trial. Rumours that blood drawn as part of the study would be sold made some people reluctant to participate in the trial. This concern about collection of children's blood for trial purposes has been widely discussed [22], [23], [27], [29], [36]. However, the perception of reduced morbidity and mortality convinced some caregivers who had initially refused IPTc to state that they would participate in an IPTc programme in future.

While issues around communication were not actively pursued in this study, the need for a single, locally-intelligible name for IPTc emerged. The French name employed in Senegal, “Prévention Saisonnière du Paludisme” (“Seasonal Malaria Prevention”) or “PSP,” effectively differentiated IPTc from curative treatment in that setting. However, the appropriateness of the French language and of this particular name would need to be explored within a broader social marketing approach before adoption of this term in Burkina Faso and Mali. A World Health Organization Technical Expert Group has recently recommended “Seasonal Malaria Chemoprevention” (SMC) as a suitable English language descriptor. While communication about the trial targeted women, our research revealed that men could play an important role both at the decision-making stage and in ensuring that their children actually received IPTc, a finding consistent with studies of IPTi [20]. More active targeting of communication about IPTc to men could help to dispel negative rumours and could also be used to encourage more active and practical male participation in the health of their children.

This study benefitted from a structured research process which allowed comparable data to be collected in two countries. We found that interviewers required a very detailed understanding of the clinical trial design and practical aspects of IPTc implementation in order to probe effectively, and that a workshop with study team physicians was an effective forum for sharing this knowledge. We found that for a number of female caregivers, expressing an opinion even on this non-sensitive, recent experience was new and challenging, while the opportunity to participate in both an interview and a FGD seemed to set participants at ease and encourage expression. Male and female CHWs expressed themselves more readily.

A key advantage of conducting qualitative research during a trial phase of a new intervention is that it provides an opportunity to ensure that there are no unchangeable aspects of the innovation that are likely to prevent its widespread adoption, and to identify potential modifications to the intervention that could maximize its uptake. Rather than attempt to dissociate perceptions from their context, this paper has focussed on understanding the perceptions of IPTc and on making specific recommendations which can be applied to future efforts to roll out the innovation.

This study has a number of limitations. Although the trial included seven sites in two countries, only limited geographical areas in these two countries were studied. While some degree of triangulation was possible, results were largely based on what participants reported, which is not always reflective of their thoughts, intentions, or future behaviour. Although participants were told that they would receive no direct benefits as a result of the interviews, association of the interviewers with the trial team may have increased participants' desire to respond positively in the hope that doing so might enable them to continue to receive free curative care. Further research will be needed regarding the experience of distributing IPTc at scale outside a trial setting and the evolution of perceptions over time, especially if high community-level coverage of IPTc over several years were to decrease not only the incidence of malaria but also the perceived threat of malaria.

### Conclusion

This study of perceptions of IPTc within the context of trials conducted in Burkina Faso and Mali provides promising indications of the potential to deliver IPTc effectively at scale. IPTc was compatible with local experiences and perceptions of childhood illness and there was no evidence that IPTc was perceived as a substitute for bed net usage, nor that it would inhibit care seeking. Trust in and respect for health and other authorities emerged as an important motivator for participation. The use of single-dose formulations could increase the speed and success of IPTc implementation at scale, as would integration and joint marketing of IPTc as part of a package of activities, such as LLIN distribution and free curative care, for which demand is already high.
